# Case Report: Secondary myelodysplastic syndrome following autologous stem cell transplantation in a patient with POEMS syndrome

**DOI:** 10.3389/fimmu.2025.1711447

**Published:** 2025-11-18

**Authors:** Miaoya Le, Nanxi Dong, Zhengwei Tan, Baodong Ye, Junfa Chen, Shuyan Liu

**Affiliations:** 1 The First School of Clinical Medicine, Zhejiang Chinese Medical University, Hangzhou, Zhejiang, China; 2 Department of Hematology, The First Affiliated Hospital of Zhejiang Chinese Medical University (Zhejiang Provincial Hospital of Chinese Medicine), Hangzhou, Zhejiang, China

**Keywords:** POEMS syndrome, myelodysplastic syndrome, autologous stem cell transplantation, secondary myeloid neoplasms, TP53 mutation, SARS-CoV-2, case report

## Abstract

This article reports a rare case of a patient with POEMS syndrome who developed secondary myelodysplastic syndrome (MDS) two years after undergoing autologous stem cell transplantation (ASCT). The patient was initially misdiagnosed with chronic inflammatory demyelinating polyneuropathy (CIDP) due to symptoms of limb numbness and weakness. Two years later, the diagnosis was corrected to POEMS syndrome. After induction therapy with the lenalidomide-dexamethasone (RD) regimen, ASCT is performed and partial remission is achieved. And lenalidomide was used for maintenance therapy. Over a year later, he was infected with SARS-CoV-2 and subsequently developed pancytopenia. Bone marrow routine revealed increased myeloblasts with multilineage dysplasia, and next-generation sequencing (NGS) found a TP53 mutation, leading to the diagnosis of secondary MDS. The pathogenesis of secondary MDS in POEMS syndrome is discussed from three aspects: cytotoxic therapy, genetic predisposition, and SARS-CoV-2 infection. This case underscores the importance of prolonged surveillance for secondary myeloid neoplasms (sMN) in POEMS patients and suggests that early genomic profiling and individualized treatment may improve outcomes.

## 1 Introduction

**Figure 1:**
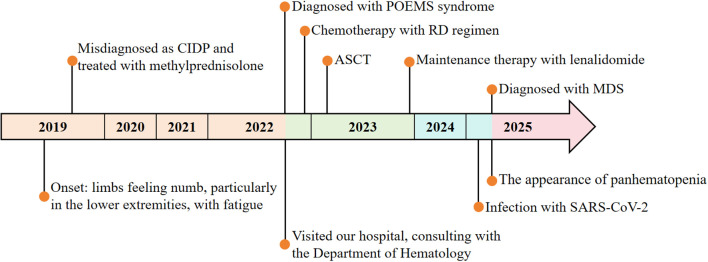
Flow chart of treatment.

POEMS syndrome is a rare paraneoplastic syndrome caused by abnormal proliferation of plasma cells, characterized primarily by polyneuropathy, organomegaly, endocrinopathy, M-protein, and skin changes. Its name is derived from the initial letters of these features (1). Although combined chemotherapy and autologous stem cell transplantation (ASCT) have markedly improved long-term survival (2), secondary myeloid neoplasms (sMN) are gaining attention as a rare late complication. This case reports a patient with POEMS syndrome who developed secondary myelodysplastic syndrome (MDS) two years after ASCT, which provides clinical evidence for the potential association between POEMS syndrome and MDS, enhances understanding of its complex complications, and offers a reference for expanding research on disease mechanisms and clinical management. The clinical course is summarized below (**Figure 1**).

## 2 Case description

**Figure 2:**
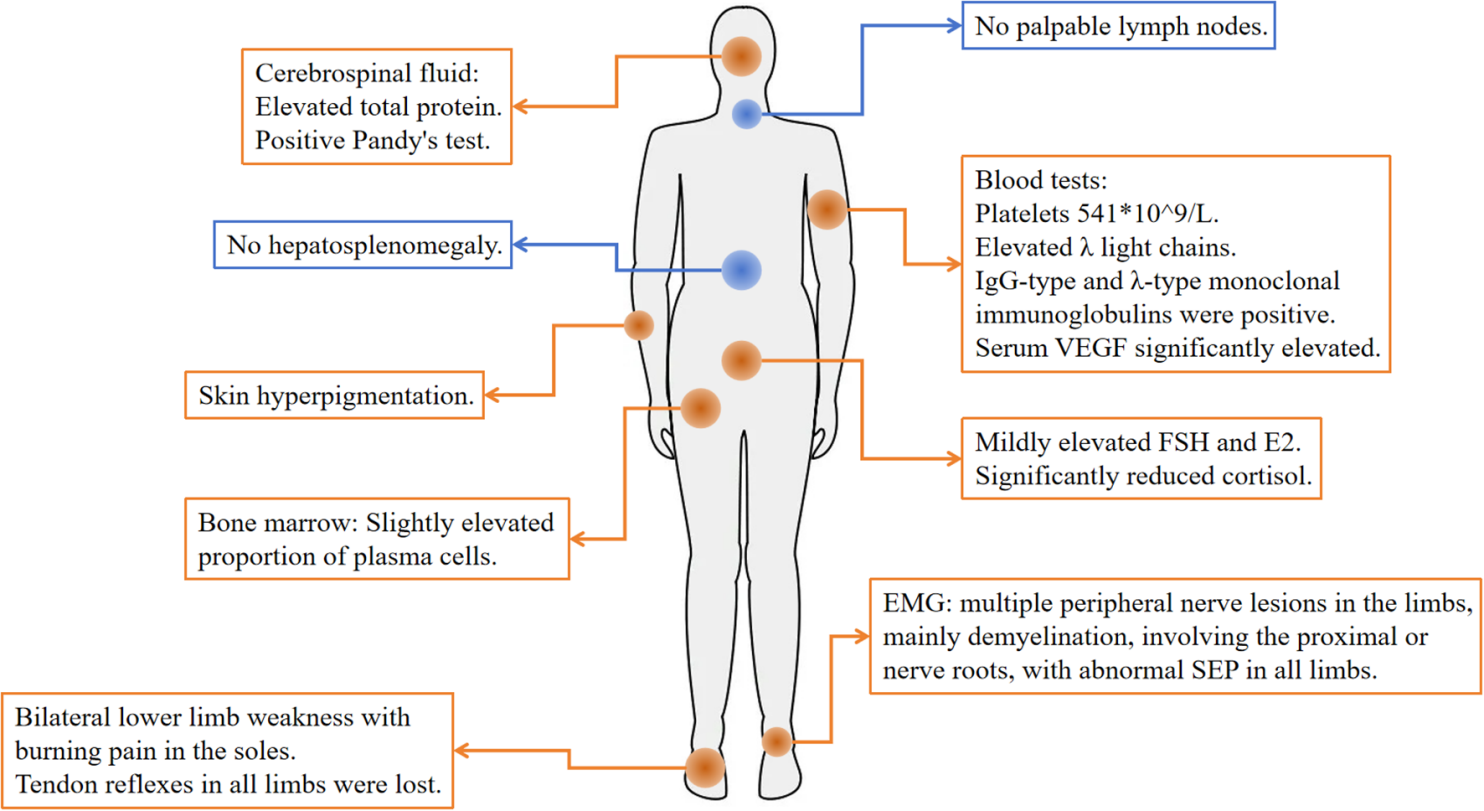
Physical examination and abnormal tests at diagnosis. Orange represents abnormal, and blue represents normal.

In October 2022, a 51-year-old male presented to Zhejiang Provincial Hospital of Traditional Chinese Medicine with recurrent numbness and weakness of all four limbs for over two years. In 2019, evaluation at the First Affiliated Hospital of Zhejiang University had included lumbar puncture, revealing cerebrospinal fluid protein elevated to 1.06 g/L, and electromyography (EMG) demonstrating motor fiber damage of peripheral nerves in both upper and lower limbs, predominantly in the lower limbs. On the basis of these findings, chronic inflammatory demyelinating polyneuropathy (CIDP) was diagnosed. The symptoms were improved significantly after treatment with methylprednisolone, but following four months of dose reduction, his condition recurred, presenting with bilateral lower limb weakness accompanied by burning pain in the soles, limiting his ambulation to 300 meters. Symptoms were attenuated by methylprednisolone escalation and exacerbated by dose reduction. On October 28, 2022, the patient visited the Department of Neurology at our hospital. Upon routine examination, he discovered an increase in light chains. After consulting with the Department of Hematology, relevant laboratory and imaging data are presented in **Table 1**, **Figure 2** and the following records.

**Figure 3:**
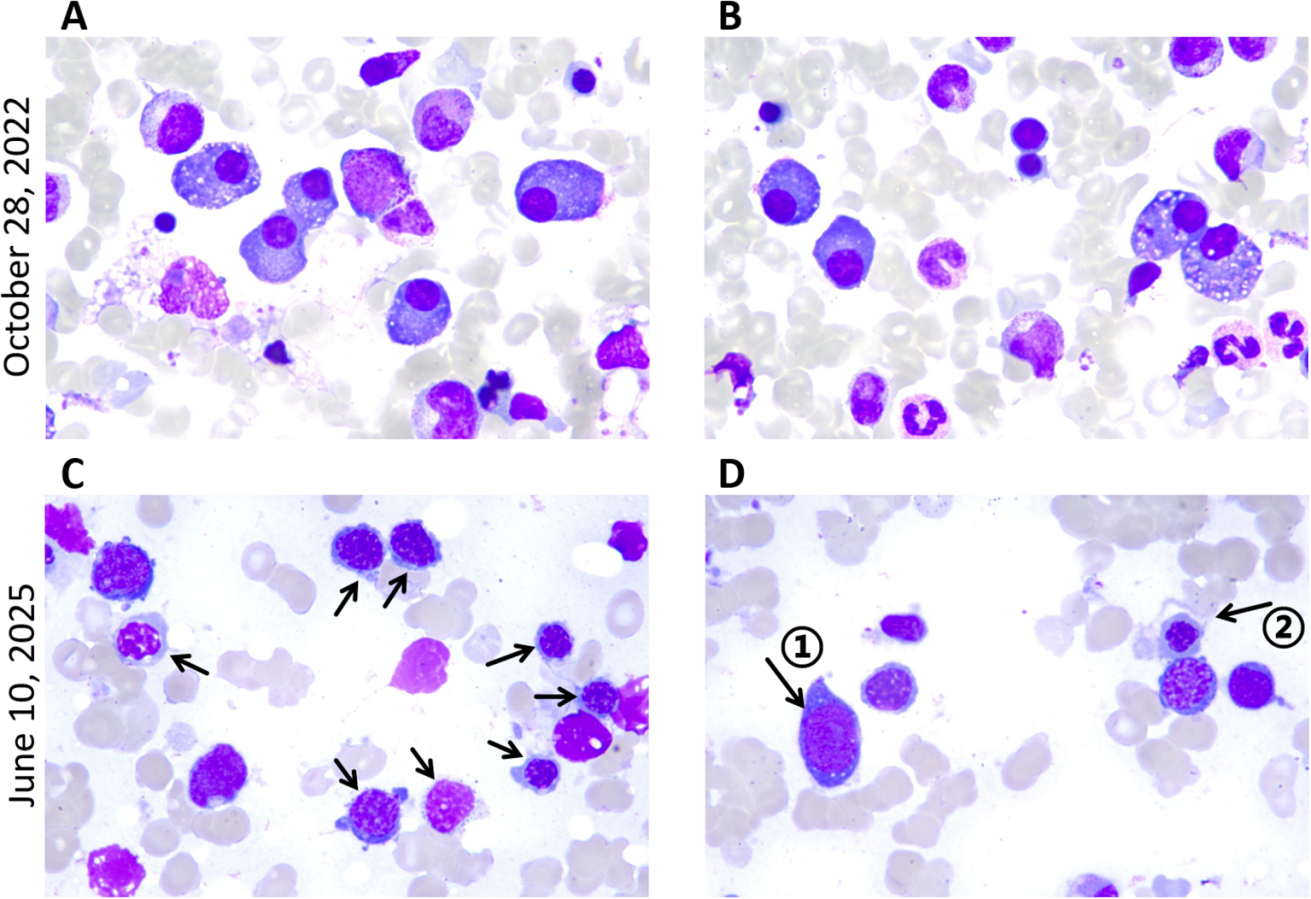
Bone marrow smears at diagnosis of POEMS syndrome and MDS. Wright-Giemsa staining of bone marrow smear from posterior superior iliac spine (magnification, x1,000); **(A, B)** High proportion of plasma cells. **(C)** Erythroid dysplasia: relatively high proportion of erythroblasts, with active erythroid hyperplasia and chromatin condensation. Arrows denote abnormal erythroblasts. **(D)** Arrow ① denotes the primitive cell, and arrow ② denotes megaloblast-like cells.

Physical examination: The patient exhibited skin hyperpigmentation without palpable lymph nodes or hepatosplenomegaly. Tendon reflexes were absent in all limbs, but muscle strength and tone were normal, bilateral deep and superficial sensation were intact, and no pathological reflexes were elicited. Relevant laboratory tests: Blood routine: white blood cells 9.7×10^9^/L, hemoglobin 149 g/L, platelets 541×10^9^/L. Biochemistry: creatinine 87 μmol/L, globulin 26.4 g/L, AST 13 U/L, ALT 15 U/L. Both serum and urine λ light chains were elevated (10.20 g/L and 12.8 mg/L), and immunofixation electrophoresis demonstrated IgG-λ monoclonal bands. Serum vascular endothelial growth factor (VEGF) was significantly elevated (800 pg/mL). Follicle-stimulating hormone (FSH) and estradiol (E2) were modestly elevated, whereas cortisol was significantly reduced (66.7 nmol/L at 8:00 AM). Adrenocorticotropic hormone (ACTH) and thyroid hormones remained within the normal range. Cerebrospinal fluid analysis showed elevated total protein (0.687 g/L), a positive Pandy test, and a normal white blood cell count. EMG showed multifocal, predominantly demyelinating, peripheral nerve lesions affecting proximal segments and nerve roots, and somatosensory evoked potentials (SEP) were abnormal in all limbs. Bone marrow routine showed a slightly elevated proportion of plasma cells (3.5%-4.5%), with preserved trilineage hematopoiesis (**Figures 3A, B**). Flow cytometry revealed abnormal plasma cells accounting for about 1.44% of nucleated cells. Cytogenetics yielded a normal 46, XY karyotype. Epstein-Barr virus (EBV) DNA and Cytomegalovirus (CMV) DNA were not detected. Ultrasonography of the thyroid, lymph nodes, liver, gallbladder, pancreas, spleen, and kidneys disclosed no abnormalities.

According to the 2014 consensus of the International Myeloma Working Group (IMWG), the patient was diagnosed with POEMS syndrome (3, 4) and classified as intermediate-risk in prognosis stratification (5). After one cycle of RD regimen (lenalidomide 25 mg qd×21d + dexamethasone 45 mg qd×d1-4, d9-12, d17-20), ASCT was scheduled. Mobilization was performed with cyclophosphamide 2.8 g×2d plus G-CSF starting February 6, 2023. On February 20, 198 mL of peripheral blood stem cells were collected, including 3.83×10^8^/kg nucleated cells, 1.76×10^8^/kg mononuclear cells, and 4.24×10^6^/kg CD34+ cells. On March 20, 2023, pre-transplant conditioning consisted of melphalan 350 mg×1d, and autologous cells were reinfused on March 22, with successful engraftment. After transplantation, persistent plantar numbness was noted, with monoclonal IgG-λ bands remaining detectable by immunofixation (**Table 1**). Because only a partial response (PR) had been achieved, lenalidomide 25 mg daily was initiated as maintenance starting December 28, 2023 (6).

In April 2025, the patient appeared with fever and diarrhea, tested positive for SARS-CoV-2 RNA, and experienced recurrent fever despite antiviral treatment. On May 8, 2025, the patient presented to our emergency department. The blood routine showed white blood cells 9.7×10^9^/L, hemoglobin 70 g/L, platelets 1065×10^9^/L, and CRP 59.01 mg/L. SARS-CoV-2 RNA remained positive, with EBV DNA, CMV DNA, and other virological tests negative. Chest CT revealed bibasilar infiltrates with bilateral pleural effusion. After inpatient antiviral therapy, the lung infection significantly improved and the virus was cleared. However, on June 7, pancytopenia was detected: white blood cells 1.2×10^9^/L, hemoglobin 50 g/L, and platelets 31×10^9^/L (**Supplementary Table S1**, **Supplementary Figure S1**). Bone marrow routine revealed 3.5% myeloblasts with multilineage dysplasia (**Table 1**; **Figures 3C, D**). Flow cytometry showed abnormal plasma cells accounting for about 0.623% of nucleated cells. Cytogenetics yielded a 46, XY karyotype, and next-generation sequencing (NGS) indicated a TP53 mutation at 79.91% variant allele frequency. Based on these findings, a diagnosis of myelodysplastic syndrome with multilineage dysplasia and low blasts (MDS-LB) was established (7) and classified as intermediate-risk according to IPSS-R (8). Subsequently, the patient underwent haploidentical allogeneic hematopoietic stem cell transplantation (allo-HSCT) and achieved successful engraftment.

**Table 1:** Partial laboratory findings.

Timepoint	2022.10 (Diagnosis)	2023.02 (Post-RD regimen)	2023.04 (Post-ASCT)	2025.04 (SARS-CoV-2 infection)	2025.05 (Emergency admission)	2025.06 (SARS-CoV-2 RNAturns negative)
White blood cell, 10^9^/L	9.7	8.6	7.4	6.5	9.7	1.2
Hemoglobin, g/L	149	137	131	90	70	50
Platelet, 10^9^/L	541	335	510	842	1,065	31
Serum λ light chains, g/L	10.2	5.76	9.06	3.05	1.96	2.15
Urinary λ light chain, mg/L	12.8	4.09	NA	32.4	52.6	64.2
Immunofixation electrophoresis	positive	positive	positive	positive	weakly positive	weakly positive
Serum VEGF, pg/ml	800	NA	NA	NA	NA	37.65
Primitive cell in bone marrow, %	1	0	NA	NA	NA	3.5
Plasma cell in bone marrow, %	3.5	1.5	NA	NA	NA	2.5
Plasma cell in flow cytometry, %	1.44	2.02	NA	NA	NA	0.623

## 3 Discussion

POEMS syndrome is a disease caused by abnormal proliferation of plasma cells, with a chronic progression. The 5-year progression-free survival (PFS) and overall survival (OS) rates are 58% and 78%, respectively (9). However, its diverse symptoms often lead to misdiagnosis. For instance, early peripheral neuropathy is often misdiagnosed as CIDP or Guillain-Barré syndrome (GBS) (10, 11). As a result, definitive diagnosis is normally delayed by 12–16 months, during which multi-organ damage and irreversible neurologic deficits may develop, even compromising survival (12, 13). In this case, CIDP was initially diagnosed in the Department of Neurology, and the correct diagnosis was not established until 2 years later.

The pathogenesis of POEMS syndrome remains incompletely understood. Although VEGF is the cytokine most strongly associated with disease activity (14), responses to anti-VEGF therapy suggest that it acts as a downstream mediator rather than the primary driver (15, 16). B-cell dysregulation is also a key factor in disease initiation. At present, anti-plasma-cell therapy is the main treatment, including ASCT, lenalidomide plus dexamethasone (RD regimen), bortezomib plus dexamethasone (BD regimen), etc., with complete response (CR) rates all exceeding 40% (2, 17, 18). Among them, ASCT shows superior efficacy in improving neuropathy, inducing more durable M-protein and VEGF responses, and prolonging PFS and OS (2).

MDS is a group of malignant clonal myeloid diseases characterized by ineffective hematopoiesis, peripheral blood cytopenia, and a high risk of progression to acute myeloid leukemia (AML) (19). It is classified as either primary or secondary. Secondary MDS accounts for 10-15% of cases (20), primarily resulting from prior exposure to cytotoxic drugs, ionizing radiation, chemical toxins, viral infections, autoimmune diseases, or malignant tumors etc (21). Therapy-related MDS and MDS arising after aplastic anemia are the most frequently recognized subtypes (22).

MDS secondary to POEMS syndrome is rarely reported, as the two disorders are assigned to distinct hematopoietic lineages (myeloid and plasma cell (B-cell)) respectively. How both lineages become clonally involved in the same patient remains unresolved. The potential reasons are discussed below under the headings of cytotoxic therapy, genetic predisposition, and viral infection.

### 3.1 Cytotoxic therapy

Due to the scarcity of sMN reports in POEMS syndrome, analysis can be conducted through homologous plasma cell disorders such as multiple myeloma (MM), Waldenström’s macroglobulinemia (WM), and monoclonal gammopathy of undetermined significance (MGUS). They are all associated with abnormally proliferating monoclonal plasma cells and produce M proteins. In MM, the incidence of secondary primary hematologic malignancies (SPHM) approaches 7%, while therapy-related myeloid neoplasms (t-MN) occur in approximately 3% (23–27). Within their treatment regimens, alkylating agents (e.g., cyclophosphamide, melphalan) and lenalidomide are considered as drivers of secondary MDS.

A study indicated that secondary acute lymphoblastic leukemia (ALL) is more frequently observed in MM patients who have not undergone ASCT, whereas sMN predominates among transplant recipients (28). The incidence of secondary MDS/AML after ASCT was approximately 1-2%, with a median interval from auto-HCT to MDS/AML diagnosis of 58.5 months (range 6.2–206.5 months) (29, 30). As early as 1970, Kyle et al. proposed that melphalan might play a role in the pathogenesis of acute leukemia (31). Current research consistently indicates that cyclophosphamide and high-dose melphalan used in pre-transplant conditioning induce mutational accumulation in myeloid cells, thereby increasing the risk of SPHM (25, 26, 32, 33). Moreover, melphalan is recognized to possess greater mutagenic potential than other alkylating agents (23).

Lenalidomide is also associated with a risk of inducing secondary malignancies (28, 34). In 2012, McCarthy et al. demonstrated that lenalidomide significantly increased the risk of secondary malignancies in MM patients after ASCT (35). A meta-analysis of seven RCTs by Palumbo et al. found an increased 5-year cumulative incidence of SPHM in lenalidomide-treated patients (3.1% vs. 1.4%) (36), with t-MN accounting for the majority at approximately 3% (37, 38). Additional studies have shown that lenalidomide significantly increases the TP53 mutation rate in t-MN patients (39–41). TP53 is a critical tumor suppressor gene, repairing DNA damage and promoting apoptosis of malignant cells. Roughly 10% of MDS patients harbor functionally deficient TP53 mutations (42). Additionally, t-MN often involves multiple genetic defects, resulting in shorter survival and poorer prognosis compared to primary malignancies, with a median overall survival of only 11.8 months from t-MN diagnosis (30, 43, 44). These findings suggest that genetic testing should be integrated into treatment planning for POEMS patients, given the potential risk of secondary malignancies.

### 3.2 Genetic predisposition

Whether sMN is attributable solely to exposure factors such as chemotherapy and radiotherapy is still a controversy. After studying secondary malignancies in plasma cell disorders, researchers found that a large number of myeloid neoplasms are diagnosed concurrently with or shortly after MM (45). Given that t-MN typically exhibits a latency period of 5–7 years (41), this finding supports the existence of an intrinsic predisposition to sMN in plasma cell disorders. Another argument is that the risk of secondary AML/MDS is increased by 11.51-fold in MM, and 8-fold in WM, despite the fact that they have divergent therapeutic approaches (46). Furthermore, MGUS without standard therapies still remains a 2.4-fold increase in the risk of developing MDS, with a median latency period of 14.4 months (47, 48). Notably, M-protein levels >1.5 g/dL confer a higher risk than those below the threshold, implicating elevated paraprotein burden as a risk factor (48). These studies collectively indicate that there are treatment-unrelated mechanisms in the progression from plasma cell disorders to myeloid neoplasms.

It has been proposed that abnormal myeloid clones already exist at the time of diagnosis or before maintenance therapy, and that continuous cytotoxic treatment selectively expands them (49–51). Mutations including TP53, TET2, DNMT3A, and ASXL1 are frequently detected before treatment, among which TP53 and TET2 showed significant expansion after sMN development, which are not only related to the pathogenesis of myeloid neoplasms, but also portend a poor prognosis (52). In this case, a high TP53 mutation had been detected at 79.91% upon MDS diagnosis. Unfortunately, the genetic testing was not performed at the time of POEMS syndrome diagnosis, so the timing of the mutation cannot be ascertained.

### 3.3 Viral infection

Multiple microbial pathogens, primarily viruses, are implicated in the pathogenesis of hematologic malignancies (53). In this case, SARS-CoV-2 infection was tested before prolonged pancytopenia, prompting speculation that the virus may have acted as an additional driver of MDS.

SARS-CoV-2 is reported to affect multiple organs, including the hematopoietic system, with both quantitative and qualitative abnormalities observed (54). Abnormalities in peripheral blood cell counts include anemia, leukopenia/leukocytosis, thrombocytopenia/thrombocytosis, etc (55). Morphologic changes mainly occur in leukocytes and platelets, including changes typically observed in MDS, such as dysplastic neutrophils and giant platelets (56). Normally, primitive cells account for 1-5% of nucleated cells in bone marrow, and they are generally absent from peripheral blood. However, there was a report of 2% primitive cells detected in the peripheral blood of COVID-19 patients (54). Nevertheless, most of these abnormalities can recover after viral clearance, and are not regarded as definitive evidence that SARS-CoV-2 directly induces MDS.

A kidney transplant recipient had been reported that developed EBV and CMV viremia following SARS-CoV-2 infection, and was diagnosed with MDS six months later (57). The authors propose that immune dysregulation induced by SARS-CoV-2 promoted clonal expansion and MDS progression, or that MDS was triggered by the reactivation of EBV or CMV. Although the immune mechanisms triggered by SARS-CoV-2 remain incompletely understood, heterogeneous activation of CD8+ or CD4+ T-cell pathways is documented, yet the selection process is unclear (58). The activated immune system produces dysregulated cytokines, thereby creating a bone marrow microenvironment conducive to clonal expansion (59, 60). Multiple viruses are implicated in the development of MDS, including CMV (61), human T-cell lymphotropic virus type 1 (HTLV-1) (62), parvovirus B19 (63), and human herpesvirus 6 (HHV-6) (64). Among them, reactivation of herpes viruses, including EBV, CMV, and HHV-6, is frequently observed in COVID-19 patients (65). An additional report describes a 30-year-old man in whom MDS was diagnosed concurrently with SARS-CoV-2 infection (66), but the causal link remains to be verified.

## 4 Conclusion

This case describes a rare clinical course of MDS secondary to POEMS syndrome after ASCT, and discusses potential reasons for the lineage transformation, including cytotoxic therapy, genetic predisposition, and SARS-CoV-2 infection. In the future, multicenter clinical studies are required to further clarify the epidemiological characteristics and risk factors for MDS secondary to POEMS syndrome, which will help improve the long-term management of patients with POEMS syndrome and may also inform prevention strategies for myeloid neoplasms secondary to other plasma cell disorders.

## Data Availability

The original contributions presented in the study are included in the article/**Supplementary Material**. Further inquiries can be directed to the corresponding author.
