# Supplementary material for: Case Report: Secondary myelodysplastic syndrome following autologous stem cell transplantation in a patient with POEMS syndrome
Source: Front Immunol. 2025 Nov 18;16:1711447. doi: 10.3389/fimmu.2025.1711447 (PMC12669161; doi:10.3389/fimmu.2025.1711447)
Supplement: Supplementary file 2 [file Table1.docx]

**Supplementary Figure 1.** Trends of white blood cells, hemoglobin and platelets from SARS-CoV-2 infection to secondary MDS diagnosis.


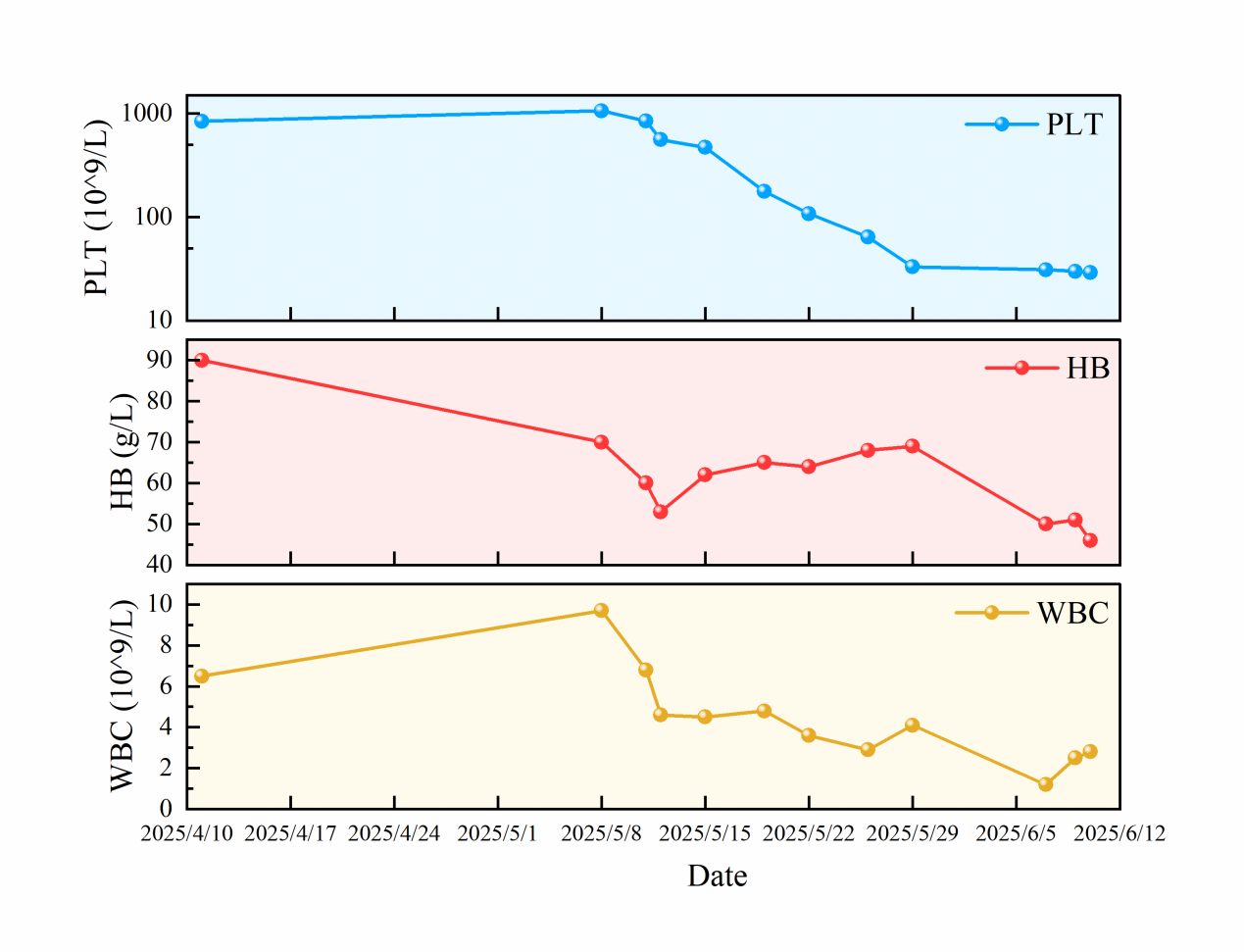


**Supplementary Table 1.** Partial laboratory findings since 2025.

| **Date** | **01.16** | **04.11** | **05.08** | **05.11** | **05.12** | **05.15** | **05.19** | **05.22** | **05.26** | **05.29** | **06.07** | **06.09** | **06.10** | **06.12** | **06.16** | **06.19** | **06.23** |
| --- | --- | --- | --- | --- | --- | --- | --- | --- | --- | --- | --- | --- | --- | --- | --- | --- | --- |
| White blood cell, 10^9^/L | 4.8 | 6.5 | 9.7 | 6.8 | 4.6 | 4.5 | 4.8 | 3.6 | 2.9 | 4.1 | 1.2 | 2.5 | 2.8 | 2.7 | 1.7 | 1.3 | 1.1 |
| Hemoglobin, g/L | 119 | 90 | 70 | 60 | 53 | 62 | 65 | 64 | 68 | 69 | 50 | 51 | 46 | 47 | 43 | 43 | 42 |
| Platelet, 10^9^/L | 201 | 842 | 1065 | 845 | 560 | 471 | 177 | 108 | 64 | 33 | 31 | 30 | 29 | 32 | 43 | 61 | 75 |
| Serum λ light chains, g/L | 3.08 | 3.05 | 1.96 | NA | NA | NA | NA | NA | NA | NA | NA | NA | NA | NA | NA | NA | 2.15 |
| Urinary λ light chain, mg/L | 29.9 | 32.4 | 52.6 | NA | NA | NA | NA | NA | NA | NA | NA | NA | NA | NA | NA | NA | 64.2 |
| Immunofixation electrophoresis | positive | positive | weakly positive | NA | NA | NA | NA | NA | NA | NA | NA | NA | NA | NA | NA | NA | weakly positive |
